# Effects of Osteopathic T9–T10 Vertebral Manipulation in Tonsillitis: A Randomized Clinical Trial

**DOI:** 10.3390/healthcare9040394

**Published:** 2021-04-01

**Authors:** Agustín Luceño-Mardones, Irene Luceño-Rodríguez, Elena Sonsoles Rodríguez-López, Jesús Oliva-Pascual-Vaca, Ignacio Rosety, Ángel Oliva-Pascual-Vaca

**Affiliations:** 1 Escuela de Osteopatía de Madrid, 28002 Madrid, Spain; agustin@osteopatiaglobal.es (A.L.-M.); joliva5@us.es (J.O.-P.-V.); angeloliva@us.es (Á.O.-P.-V.); 2 Centro Sanitario de Fisioterapia y Osteopatía Agustín Luceño, 10005 Cáceres, Spain; 3 Irene Luceño Psycho-Sexology Center, 28200 Madrid, Spain; iluceno@ucm.es; 4 Department of Physiotherapy, Universidad Camilo José Cela, 28692 Madrid, Spain; 5 Departamento de Fisioterapia, Universidad de Sevilla, 41004 Sevilla, Spain; 6 Escuela Universitaria Fco. Maldonado, Osuna, 41640 Sevilla, Spain; 7 School of Medicine, University of Cadiz, 11003 Cádiz, Spain; ignacio.rosety@uca.es

**Keywords:** tonsillitis, osteopathy, manual therapy, physical therapy, spinal manipulation, OMT, otorhinolaryngology, otolaryngology, tonsillectomy

## Abstract

This study aimed to determine whether osteopathic manipulation of the T9–T10 vertebrae improves the evolution of tonsillitis. A randomized, stratified, controlled clinical trial with blinded patients, evaluator and data analyst was performed. The patients in the control group (CG) underwent a “sham” manipulation. A high-speed, low-amplitude technique was applied to the T9–T10 vertebrae in the osteopathic manipulative group (OMG) patients. The number of days needed to resolve the tonsillitis was significantly lower (*p* = 0.025) in the OMG (2.03 ± 0.95 days) than the CG (2.39 ± 0.82 days). Additionally, the number of episodes of tonsillitis after the treatment decreased significantly more in the OMG (0.8 ± 1.88 episodes/year in total) than the CG (2 ± 2.12) (*p* = 0.005). In the OMG, 60.8% had no recurrences of tonsillitis, compared to 22.5% of the CG, in the following year (χ^2^ (1) = 15.57, *p* < 0.001). No patients reported adverse effects. It has been concluded that during an episode of tonsillitis, the number of days to resolution was significantly lower after the application of an osteopathic manipulation of the T9–T10 vertebrae, compared to a sham manipulation. The number of subsequent year tonsillitis episodes was greatly reduced in both groups, significantly more in the OMG than in the CG patients.

## 1. Introduction

Palatal tonsillitis, either acute, recurrent or chronic, is a relatively common disease [1], especially in childhood [1,2]. The treatment of choice is the use of nonsteroidal anti-inflammatory drugs (NSAIDs) and/or antibiotics [1,3,4], and in some cases tonsillectomy [5,6,7,8,9]. These treatments can present side effects [5,6,10,11,12,13] in the short and long term [14]. Each episode of tonsillitis also implies costs related to absence from work and school [1,9,10,15]. With tonsillectomy, adults experience a sore throat for an average of 13 to 17 days [10,12]. In addition to being painful [9,10], the risk of iatrogenic morbidity [6,7,9,10,16,17] and mortality [5,6,16] of tonsillectomy must be taken into account. Although tonsillectomies have decreased in recent decades [18] (except for cases of sleep apnea due to hypertrophic tonsils and recurrent tonsillitis) [6,7,19,20], it is still one of the most frequent surgical interventions [6,9,10,15]. Tonsillectomy rates vary greatly between countries [18]. Among alternative and complementary medicine interventions, spinal manipulations have been used to treat tonsillitis. Through occiput-cervical manipulation, a favorable evolution has been obtained in recurrent or chronic childhood tonsillitis, in which a high rate of pathological blockages of joints was also found [21]. This favorable evolution might be due to the fact that spinal manipulation may influence the biomarkers of local and systemic inflammation [22,23]. However, cervical manipulations are considered riskier manipulations, although severe complications are very rare [24,25,26,27]. Manipulation of the thoracic vertebrae presents fewer severe adverse events than cervical manipulation [25,27]. Furthermore, the vertebral segments T9 and T10 innervate the adrenal glands [28,29,30,31], which produce cortisol, and patients with tonsillitis have been found to have altered cortisol levels [32]. This hormone may influence the immune response [22,33,34], and its levels are modified after thoracic manipulation [22,23,35,36]. Thus, in a prospective study (75 participants) without a control group, manipulation of the lower thoracic vertebrae (mainly in the T9–T10 segments), obtained 55% resolution sooner than 24 h and 76% sooner than 48h, showing promising results for treating tonsillitis in both children and adults [37]. The objective of the present study is to determine if osteopathic manipulation of the T9–T10 vertebrae improves the evolution of tonsillitis, under the hypothesis that it produces a decrease of days with symptoms and a decrease in recurrences. In addition, it is intended to analyze the possible influencing factors.

## 2. Materials and Methods

### 2.1. Design

A stratified, randomized placebo trial was conducted with blinded patients, evaluator and data analyst. The study was carried out between 2-21-2014 and 6-30-2018 in the private center of Physiotherapy and Osteopathy of the main researcher of the study, in the city of Cáceres (Spain). The study was prospectively registered (ACTRN12612000068864), it followed the principles of the Declaration of Helsinki in its latest version [38] and was approved by the Ethics Committee of the University of Seville.

### 2.2. Participants

The participants were recruited from a Physiotherapy and Osteopathy Health Center, a pediatrician’s office, a General Medicine office, and a private clinic (in its Emergency Service and in a General Medicine office). Independently of the place where the recruitment took place, the participants were diagnosed by a collaborating physician who also visually measured the tonsil at enrollment in order to classify it in degrees (0-IV) according to the occupation of the oropharyngeal space [2]. Participants of both sexes between 3 and 65 years old diagnosed with acute or recurrent tonsillitis of less than 48 h of evolution, or chronic tonsillitis in the symptomatic phase, were included in the study. Those vaccinated or treated with immunomodulators (immunoferon and the like) [39] during three previous years to recruitment, any subject who was suffering an episode of pharyngitis or adenoiditis without palatal tonsillitis (i.e., tonsillectomized), being treated with antibiotics immediately before the tonsillitis episode, subjects who did not provide a phone number in the initial questionnaire for control calls and subjects who presented contraindications to the experimental treatment [40,41] were excluded. Before the start of the study, all participants or their parents or guardians in case of minor signed an informed consent form.

### 2.3. Treatment Protocols

**Figure 1:**
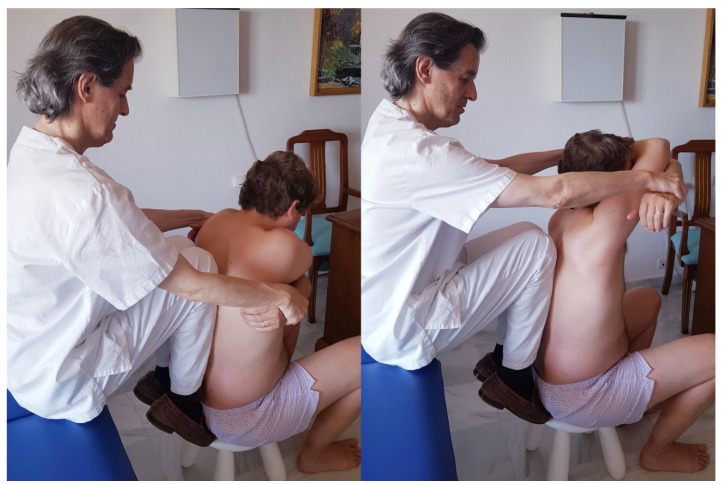
Placebo maneuver and its development.

The subjects were randomly assigned to the study groups by using two computer-generated (Microsoft Excel) tables of sequence of numbers (2:1 ratio experimental/control), one table for those participants treated with antibiotics and the other one for those who were not, in order to avoid between-groups differences related to antibiotic use. The randomization sequence was guarded by an independent collaborator who guaranteed its concealment. To implement random allocation, sequentially numbered opaque sealed envelopes were used. Those researchers and collaborators who recruited the sample were blinded to the number sequence and to the intervention assignment. Additionally, every intervention was blinded for both the participants and evaluators. The patients in the control group (CG) underwent sham manipulation, consisting of a careful 150° passive flexion of the shoulders, with gentle contact of the osteopath’s knees in the middle thoracic vertebrae, without impulse or causing tension. (Figure 1)

**Figure 2:**
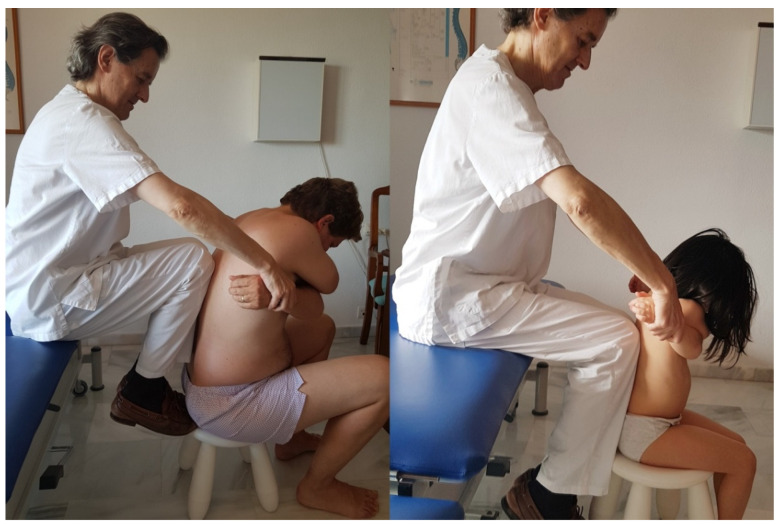
T9–T10 thrust manipulation in sitting position (adult man and 5-year-old girl).

Patients in the osteopathic manipulation group (OMG) had a high-speed, low-amplitude technique applied to the T9–T10 vertebrae in a sitting position, with crossed arms, with contact via the therapist’s knees [42,43,44] (Figure 2).

The osteopathic manipulative procedure or the sham manipulation were only applied once, as soon as possible after the enrollment, always in the first 48 h of the beginning of the symptoms of that episode of tonsillitis. The same physiotherapist—osteopath, with professional experience of more than 20 years, applied the interventions to both groups. This researcher did not participate in the recruitment, randomization, evaluation nor statistical analysis, being blinded to all of those processes.

Regardless of the application of the experimental procedure or placebo, all subjects continued pharmacological treatment (analgesics, NSAIDs and/or antibiotics) prescribed by their doctor.

### 2.4. Evaluations

The main results were the number of days for the total resolution of the symptoms of tonsillitis (fever, sore throat, cough, etc.) and the number of episodes of tonsillitis during the following year. Both were measured by telephone consultation [45,46,47,48]. The number of days for the resolution of the symptoms was evaluated seven days after the application of the experimental treatment or the placebo maneuver, and the result was also classified as excellent (resolution in less than 24 h), good (resolution in less than 48 h), moderate (improvement from the first day, but with resolution ≥48 h) or poor (no improvement on the first day, with resolution ≥48 h). The number of recurrences during the following year was evaluated through monthly telephone calls for 12 months. In the case of those participants younger than 18 years old, all the information was given by their parents or tutors. As secondary results, the different associations between the variables measured were evaluated. The independent variables were collected through the initial questionnaire filled out by the patient and the initial clinical examination carried out by the collaborating physician. In addition to the outcome variables, age, gender, season of the year, degree of tonsillar hypertrophy, consumption of NSAIDs, paracetamol and prescribed antibiotics were recorded. Additionally, we also registered the number of episodes in the two years prior to the study, the scheduling of tonsillectomies, the presence of a fever, odynophagia, cough, pultaceous tonsillitis, mucus the previous days, ear pain or infection, habitual nasal voice, nasal voice during the episode, habitual snoring, snoring during the episode or adenitis greater than 2 cm.

From the moment that the participants were included in the study, they were adverted about the information they would be asked by phone, so that they had to pay attention and control it. For instance, they were asked to assess any sign of hyperthermia thermometrically. In the case of participants younger than 18 years old, all of this was explained to their parents or tutors.

### 2.5. Statistical Analysis

Statistical analysis was carried out with SPSS 22.0 software (SPSS Science, Chicago, IL, USA). The descriptive study of the variables was carried out in tables with mean, standard deviation, 95% confidence interval (CI) for continuous variables and in percentages for qualitative variables. Before carrying out the statistical analysis, the conditions of its application were considered; the Shapiro–Wilk test was used to verify that the sample met normality criteria. The Mann–Whitney U test was used to verify homogeneous distribution between groups when they did not meet normality criteria; otherwise, the T test was used. Chi-square was used for qualitative variables. A least squares estimation was used to quantify the interval of difference between groups. Analysis of variance of repeated measures (ANOVA) with linear model with Bonferroni adjustment was used to test the profile of the change in the result of the number of episodes in the two and one year before and after the two study groups, and the comparison in pairs according to time and group. The global clinical effects for the repeated measures analysis were calculated using the Eta-squared value (η2), categorized as small = 0.01, medium = 0.06 and large = 0.14 [49]. For the analysis of the number of days of resolution, the Mann–Whitney U test was used. Pearson’s chi-square was used for the analysis according to group of the resolution greater than 48 h, initial result and at 12 months. The bivariate correlations of the quantitative variables were analyzed using Pearson’s coefficient. A significance level *p* < 0.05 and a confidence level of 95% were established. Finally, participants under 18 years old were considered as children.

The sample size calculation was performed (GRANMO v7.12; IMIM Hospital del Mar–Barcelona–Spain) for the proportion of cases resolved in the first 48 h after the application of the intervention. An alpha level of 0.05 and a desired power (beta) of 80% with a bilateral contrast were assumed. Additionally, the sample size was estimated according to an expected proportion of resolutions of 65% in the first 48 h in the OMG and a proportion of 36% in the CG. Losses were estimated at 15%. These assumptions generated a sample size of 80 participants in OMG and 40 in CG.

## 3. Results

### 3.1. Sample

**Figure 3:**
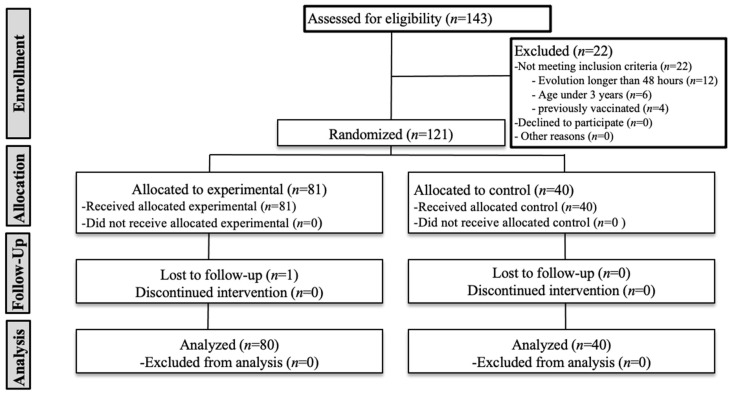
Consort flow diagram.

While all of the 40 subjects randomized to CG completed the study, one of the eighty-one subjects randomized to OMG never answered the phone calls, so they were considered as a withdrawal. (Figure 3)

Thus, 120 subjects (70 women) aged between 3 and 57 years (23.53 ± 14.84 years) completed this study. No between-groups differences were found for any of the baseline characteristics (*p* > 0.05 for all variables). (Table 1).

Comparing the number of episodes two years before with that of the previous year, there was a significant increase in OMG (*p* = 0.011) and a clear similar trend in CG (*p* = 0.052). This increase occurred both in children (with a change from 4.79 episodes two years earlier, to 6.10 in the previous year) and in adults (from 4.19, to 4.94). There was a negative correlation between age and the number of episodes in the previous year (r = −0.186; *p* = 0.042). Tonsillar hypertrophy was more prevalent in males (*p* = 0.035), in those under 18 years of age (*p* = 0.009) and among those who snored during the episode (*p* = 0.047). As for the pediatric sample, when compared with the adult participants, it had a higher male composition (*p* = 0.033), had a higher prevalence of nasal mucus (*p* = 0.004) and snoring during episodes (*p* = 0.018).

### 3.2. Intervention Effects on Tonsillitis

The number of days needed to resolve the tonsillitis was significantly lower (PSest = 0.247; *p* = 0.025) in the OMG (2.03 ± 0.95 days) than the CG (2.39 ± 0.82 days). In the case of the adults, this significant difference persisted (PSest = 0.263; *p* = 0.001), with better results for the OMG (2.00 ± 0.81 days) compared to the CG (2.44 ± 0.61 days), but not in the pediatric participants (*p* = 0.856; OMG 2.00 ± 1.00 days; CG 1.91 ± 0.83 days).

The 63.75% of the subjects of the OMG resolved the episode in less than 48 h, compared to 42.5% in the CG (Table 2). In the case of adults, 68.6% of the subjects of the OMG resolved the episode in less than 48 h, compared to 33.3% in the CG, showing a significant difference with medium effect size, which was not present in children.

**Figure 4:**
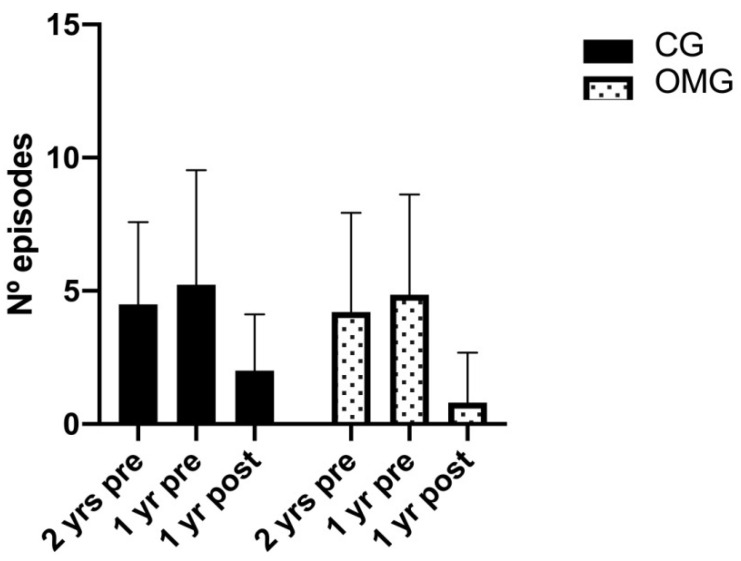
Number of episodes of tonsillitis two years before, one year before and one year after the study, in the CG and the OMG.

The number of episodes of tonsillitis after intervention decreased in the CG and in the OMG, as shown in Table 3 and Figure 4. Ten tonsillectomy cases (two from the CG and eight from the OMG), six cases that were vaccinated (three of the CG and three of the OMG) in the following year by their doctor and three cases of the OMG that had not registered the number of episodes of the second previous year were excluded.

In the analysis of the number of post-treatment episodes according to age, the OMG achieved a significant improvement when compared to the control group both in adults (PSest = 0.25; *p* < 0.001; OMG 0.69 ± 1.61 episodes; CG 2.00 ± 2.07 episodes) and in children (PSest = 0.29; *p* = 0.026; OMG 0.92 ± 2.26 episodes; CG 2.00 ± 2.33 episodes).

In the supporting repeated measures ANOVA including data points from two years before, one year before and after intervention, we did not find a significant time by group interaction effect (F (2,198) = 1.408, *p* = 0.247, η2 = 0.014). However, we found a significant time interaction effect (F (2,198) = 82.897, *p* < 0.001, η2 = 0.456). Specifically, post hoc analysis in both groups showed significant between-time differences (*p* < 0.001) in the change from two years and one year before intervention compared to after the intervention. Furthermore, the number of episodes of tonsillitis after the treatment decreased significantly more in the OMG (0.8 ± 1.88 episodes in total) than the CG (2 ± 2.12) (*p* = 0.005).

The 60.8% of the OMG had no tonsillitis recurrences, against 22.5% of the CG, in the following year (χ^2^ (1) = 15.57, *p* < 0.001), as shown in Table 4.

In the CG, there was a significant correlation (*p* = 0.012) between the number of episodes in the previous year and recurrence at 12 months, but not in OMG (*p* = 0.095). Additionally, there was no correlation between age and the number of episodes in the subsequent year (*p* = 0.950). Five subjects from the OMG and one from the CG did not end up receiving the planned tonsillectomy, due to the fact that they had presented an average of 10 episodes in the previous year. Five of them were hypertrophic.

No patients reported adverse effects, not even soreness, in relation to the manipulation received, neither in the OMG nor in the CG.

## 4. Discussion

In spite of the importance of results from scientific research in evidence-based practice for osteopaths [50], there are not many studies about the role of spinal manipulation in tonsillitis, as previously explained [21,37].

Our study’s main objective was to explore if T9–T10 osteopathic manipulation reduces the duration of symptoms as well as the number of recurrences in subjects with tonsillitis. Through a randomized clinical trial, we observed that the number of days of resolution of the episode of tonsillitis was significantly lower in the OMG than in the CG. This result was only found in adults but not in children. Likewise, the number of episodes of tonsillitis in the following year decreased significantly in both groups, compared to the previous year, but significantly more in the T9–T10 manipulation group. In this case, this effect was found in both adults and children. It should be noted that the follow-up comprised one whole year. Furthermore, there were no intergroup differences at baseline for any variable, including taking antibiotics. However, the data obtained about the number of episodes in the previous year and, even more so, two years earlier, may be subject to recall bias. Previous studies reported an overestimation of the frequency of sore throats by parents in retrospective-prospective studies during childhood [51]. What is more, it has to be taken into account that children younger than three were excluded due to the low number of tonsillitis diagnosed in the first year of life, which would affect the reported data for the previous years. On the other hand, the great improvement in the number of episodes in the subsequent year in both groups may be influenced by “attention bias” or Hawthorne effect [52], since the participants received a monthly telephone call from the collaborating nurse, and they knew they belonged to a clinical trial in which they had received treatment. Regarding the weight of the intervention group with a 2:1 randomization, it was due to ethical issues and the existence of indications of promising results for the experimental intervention [37]. Finally, in spite of the fact that the phone follow-up is a reality in both clinical and research settings [45,46,47,48], it is a limitation of the study that, in that it is based on subjective outcomes of patients’ interview through telephone.

After childhood, there is a natural tendency for the number of episodes to decrease [1,53], but in our study, no such trend was found in the previous two years, neither in children nor in adults. In contrast, the number of episodes of tonsillitis increased from the two years before to the year before participation in the study. This increase was significant in the OMG, although not in the CG (*p* = 0.052), perhaps due to the smaller size of the CG. While the influence of recall bias cannot be ruled out, this increasing trend in tonsillitis episodes in the previous two years indicates that there was no decreasing trend in tonsillitis episodes, either natural or due to the treatments received. Therefore, the large decrease in episodes in the subsequent year observed in both groups does not seem attributable to the natural course of the disease or to the conventional treatments applied to the subjects in the sample. Furthermore, it should be remembered that vaccination and the consumption of immunomodulators [39] the previous year were considered as exclusion criteria. Although there was a negative correlation between age and the number of episodes in the previous year, this correlation was small (r = −0.186), and there was no correlation with the number of episodes in the following year (*p* = 0.950). Therefore, we can hypothesize that age had little influence on the long-term result and, therefore, treatment can be considered from three years of age.

Several studies on tonsillitis describe a decrease of 0.5 to 1 episode on average in the subsequent year of follow-up in the control group, as shown in a recent clinical practice guideline [2]. In a study with children and adults, the placebo group obtained 51% of the cases without relapses in the following year [4,54], although in our CG there was a 22.5% of cases without relapses. In our sample, the considerable decrease in post-intervention episodes in CG led us to think that there could be a strong placebo component in both study groups [55,56,57]. In one pediatric study about prophylaxis with cefpodoxime versus placebo [4,58], the number of acute episodes at 12 months was reduced by 84% in the intervention group compared to 15% in the placebo group. In our sample, the reduction in the number of episodes of CG infants was much greater (64% fewer episodes, versus 83% reduction in OMG). This makes us suppose a strong placebo effect in pediatric age in our study, which might be increased by the fact that the parents/tutors observed the application of the procedures. Additionally, it could make the parents/tutors more likely to report a positive outcome. What is more, before the treatment, patients generally could already be perceived as very hopeful, possibly because the study center has been involved in research on this issue for years, with hundreds of cases treated in the region, which could favor the placebo effect [59]. To this effect, the aforementioned recall bias [51] can be added, which hypothetically would overestimate the number of episodes in the previous years, while those in the following year would be better quantified by monthly follow-up calls. In any case, regardless of the possible placebo effect of both groups, the number of post-treatment episodes in the OMG was significantly lower compared to the CG.

In the same way, although the OMG obtained a significantly faster total resolution of symptoms than the CG for the current episode, the good short-term results in a number of CG cases could be partly explained by this placebo effect as well [60]. However, taking up to two days for the episode to evolve, drug treatment and spontaneous remission can also explain these data. A Cochrane analysis [3] showed that spontaneous healing occurred in adults on day three in approximately 40%, and that the antibiotic group healed on average 16 h (0.67 days) earlier than the placebo group (in our sample, 0.36 days formerly the OMG). In clinical practice guidelines [1] it is considered that with adequate therapy, most patients, especially adolescents and adults, are asymptomatic within 48 h (in our study they were 63.75% of the OMG).

Although the objective of our study was not to analyze the possible mechanisms of action that could explain the results obtained, we think that the neuroendocrine pathway should be considered. However, we did not evaluate neuroendocrine data, which constitutes a limitation of the study. As previously explained, patients with tonsillitis show altered cortisol levels [32], which is known to influence the immune response [22,33,34]. The adrenal glands, which produce cortisol, are innervated by T9 and T10 levels [28,29,30,31]. Furthermore, spinal manipulation may influence the biomarkers of local and systemic inflammation [22,23], and specifically, thoracic manipulation modifies cortisol levels [22,23,35,36]. All of this could influence the evolution of tonsillitis. The correlation observed in CG between the number of episodes in the previous year and recurrence at 12 months did not occur in the OMG, which leads us to hypothesize that manipulative treatment may be effective regardless of the number of previous episodes. In other words, a high number of episodes in the previous year is a poor prognostic factor for recurrence in the natural course of tonsillitis, but not after the proposed osteopathic treatment. The fact that six planned tonsillectomies were avoided (five in the OMG), despite having presented 10 episodes on average in the previous year and that five of them were hypertrophic, suggests that manipulative treatment might be proposed prior to decision making or performance of tonsillectomy, being able to prolong the expectant attitude in some nonurgent cases [2,9,10], even when the number of previous episodes or the tonsil size suggest a possible poor evolution. This is supported by the fact that, without adenoid hypertrophy, the size of the palatine tonsils does not correlate with obstructive sleep apnea syndrome [2].

In our study, no distinction was made between viral and bacterial pharyngo-tonsillitis. However, whether the doctor had prescribed antibiotics or not was controlled, either due to antistreptolysin O titer (ASOT), clinical suspicion of bacterial infection [38] or by confirmation by rapid antigen detection tests of GABHS or pharyngeal exudate culture. In future studies, it would be interesting to perform rapid antigenic detection tests of GABHS, ASOT titer and/or throat swab culture [53] on all participants to find out if the results of T9–T10 manipulation are influenced by the etiology of tonsillitis (viral or bacterial).

## 5. Conclusions

During an episode of tonsillitis, the number of days to resolution was significantly lower after the application of an osteopathic manipulation of the T9–T10 vertebrae compared to the application of a sham manipulation. In most OMG patients, remission of tonsillitis symptoms was obtained in less than 48 h, but not in CG. In addition, the number of tonsillitis episodes in the following year was greatly reduced in both groups, significantly more in OMG than in sham patients. More studies are needed to confirm these results and, if confirmed, to analyze the possible mechanisms of action.

## 6. Declaration

Ethical approval: This clinical trial was approved by the Ethical Committee for experimentation of the University of Seville, Seville. Informed consent was obtained from all participants prior to enrollment in the study, and all rights were protected. Transcriptions were anonymized and treated with strictest confidence. All identifying information was removed by giving each participant a unique code that was used to attribute comments during analysis. The protocol was performed following the Ethical Principles for Medical Research in Humans of the Declaration of Helsinki.

This trial was registered in the ANZCTR (ACTRN12612000068864).

## Figures and Tables

**Table 1:** Baseline characteristics of participants.

		Control Control Group		Osteopathic Manipulative Group		
Characteristics		Mean/*n*	SD/%	Mean/*n*	SD/%	*p*-Value
Age (years) (*n* = 120)		25.30	15.11	22.64	14.73	0.439
N° episodes 2 years before (*n* = 116)		4.75	3.95	4.21	3.61	0.435
N° episodes 1 year before (*n* = 120)		5.45	4.13	5.16	3.89	0.781
Sex (*n* =120)	Male	17	42.5%	33	41.3%	0.896
	Female	23	57.5%	47	58.8%	
Fever (*n* =120)	No (Temperature <37.5 °C)	10	25.0%	21	26.3%	0.883
	Yes (Temperature ≥37.5 °C)	30	75.0%	59	73.8%	
Pain or odinophagy (*n* = 119)	No	0	0.0%	4	5.1%	0.148
	Yes	40	100.0%	75	94.9%	
Cough currently (*n* = 118)	No	22	55.0%	32	41.0%	0.149
	Yes	18	45.0%	46	49.0%	
Levels of tonsilar hypertrophy (*n* = 115)	The pillars do not protrude	1	2.6%	5	6.7%	0.760
	Occupy less than 25% of the space	9	23.1%	16	21.3%	
	Occupy 25–49% of the space	12	30.8%	20	26.7%	
	Occupy 50–74%	14	35.9%	31	41.3%	
	Occupy >75%	3	7.7%	3	4.0%	
Mucus days before the episode (*n* = 119)	No	16	40.0%	28	35.4%	0.627
	Yes	24	60.0%	51	64.6%	
Pain or infection of ears (*n* = 119)	No	25	64.1%	47	58.8%	0.575
	Yes	14	35.9%	33	42.3%	
Usual nasal voice (*n* = 119)	No	32	80.0%	58	73.4%	0.429
	Yes	8	20.0%	21	26.6%	
Nasal voice in the episode (*n* = 119)	No	6	15.0%	11	13.9%	0.874
	Yes	34	85.0%	68	86.1%	
Snore usually (*n* = 117)	No	22	56.4%	56	71.8%	0.096
	Yes	17	43.6%	22	29.2%	
Snore during the episode (*n* = 116)	No	11	28.2%	30	39.0%	0.252
	Yes	28	71.8%	47	61.0%	
Take prescribed antibiotics (*n* = 120)	No	18	45.0%	37	46.3%	0.897
	Yes	22	55.0%	43	53.8%	
Take prescribed NSAID (*n* = 119)	No	6	15.0%	22	27.8%	0.119
	Yes	34	85.0%	57	72.2%	
Take prescribed paracetamol (*n* = 120)	No	15	37.5%	38	47.5%	0.298
	Yes	25	62.5%	42	52.5%	
Pultous tonsilitis (*n* = 117)	No	24	61.5%	49	62.8%	0.893
	Yes	15	38.5%	29	37.2%	
Palpable adenitis over 2 cm (*n* = 116)	No	5	13.2%	21	26.9%	0.095
	Yes	33	86.8%	57	73.1%	
Station (*n* = 120)	Spring	8	20.0%	13	16.3%	0.673
	Summer	8	20.0%	12	15.0%	
	Autumn	9	22.5%	26	32.5%	
	Winter	15	37.5%	29	36.3%	

SD, standard deviation; NSAID, non-steroidal anti-inflammatory drugs.

**Table 2:** Distribution of the results in the first week (E = Excellent, G = Good, M = Moderate and P = Poor) in relation to the group to which they belonged (CG = Control Group, EG = Experimental Group) and according to age.

	Results 1st Week				Results 1st Week Grouped	
Group	E	G	M	P	E + G (Resolution < 48 h)	M + P (Resolution ≥ 48 h)
CG	5 (12.5%)	12 (30.0%)	14 (35.0%)	9 (22.5%)	17 (42.5%)	23 (57.5%)
EG	20 (25.0%)	31 (38.75%)	11 (13.75%)	18 (22.5%)	51 (63.75%)	29 (36.25%)
*p* value	χ^2^ (3) = 8.35, *p* = 0.039, V = 0.264				χ^2^ (1) = 4.90, *p* = 0.022, V = 0.202	
**Children**						
CG	4 (30.7%)	4 (30.7%)	4 (30.7%)	1 (7.7%)	8 (61.5%)	5 (38.5%)
EG	9 (31%)	7 (24.1%)	5 (17.2%)	8 (27.6%)	16 (55.2%)	13 (44.8%)
*p* value	χ^2^ (3) = 2.57, *p* = 0.462				χ^2^ (1) = 0.15, *p* = 0.700	
**Adults**						
CG	1 (3.7%)	8 (29.6%)	10 (37%)	8 (29.6%)	9 (33.3%)	18 (66.6%)
EG	11 (21.5%)	24 (47%)	6 (11.7%)	10 (19.6%)	35 (68.6%)	16 (31.4%)
*p* value	χ^2^ (3) = 11.23, *p* = 0.011, V = 0.38				χ^2^ (1) = 8.94, p = 0.003, V = 0.34	

E (resolution in <24 h); G (resolution in <48 h); M (improvement on the first day, resolution ≥48 h); P (no improvement on the first day, resolution ≥48 h).

**Table 3:** Analysis of covariance for efficacy of intervention: number of episodes of tonsillitis.

	Control Group (*n* = 35)		Osteopathic Manipulative Group (*n* = 66)				
	Mean	SD (95% CI)	Mean	SD (95% CI)	*p*-Value (Time)	*p*-Value (Group Time)	*p*-Value (Time)
Two years before	4.49	3.09 (3.19–5.78)	4.20	3.73 (3.25–5.14)	0.721	0.247	<0.001
One year before	5.23	4.30 (3.90–6.55)	4.86	3.76 (3.89–5.83)	0.660		
After intervention	2	2.12 (1.33–2.66)	0.80	1.88 (0.32–1.28)	0.005		
Two years before vs. after intervention	−2.48	3.59 (−3.84–−1.12)	−3.39	3.15 (−4.38–−2.40)	CG *p* < 0.001; OMG *p* < 0.001		
One year before vs. after intervention	−3.22	3.52 (−4.55–−1.89)	−4.06	3.06 (−5.02–−3.09)	CG *p* < 0.001; OMG *p* < 0.001		

SD, standard deviation; CI, confidence interval; CG, control group; OMG, osteopathic manipulative group.

**Table 4:** Analysis of covariance for efficacy of intervention: tonsillitis recurrence at 12 months.

	Control Group (*n* = 40)		Osteopathic Manual Group (*n* = 79)			
	Count	%	Count	%	*p* Value (Exact Sig. 2-Sided)	Chi-Square
Recurrence	31	77.5%	31	39.2%	<0.001	χ^2^ (1) = 15.57
No recurrence	9	22.5%	48	60.8%		

## Data Availability

The data presented in this study are available on request from the corresponding author.
